# Route of Surgery for Sentinel Node Biopsy in Endometrial Cancer: Laparoscopy Versus Robotics

**DOI:** 10.3390/jcm14124013

**Published:** 2025-06-06

**Authors:** Angela Fierro, Isabel Flores, Irene Pellicer, Maria Alonso-Espias, Virginia Garcia-Pineda, Ignacio Zapardiel, Myriam Gracia

**Affiliations:** 1 Gynecologic Oncology Unit, La Paz University Hospital, 28046 Madrid, Spain; angelafierro117@gmail.com (A.F.); mariaalonsoespias@gmail.com (M.A.-E.); virginia.garciapineda@gmail.com (V.G.-P.); dra_gracia@hotmail.com (M.G.); 2 Gynecologic Oncology Unit, La Linea University Hospital, 11300 Cadiz, Spain; isabelflores23496@gmail.com

**Keywords:** endometrial cancer, sentinel node biopsy, indocyanine green, minimally invasive surgery

## Abstract

**Background/Objective**: Sentinel lymph node (SLN) mapping is an accepted technique for the nodal staging of early-stage endometrial cancer. It is carried out commonly by minimally invasive approach, either by laparoscopy or robotics-assisted surgery. The primary aim of this study was to compare the detection rate of SLN mapping between laparoscopic and robotic surgery. **Methods**: A retrospective observational study including patients operated on from February 2024 to March 2025, diagnosed with endometrial cancer who underwent hysterectomy, bilateral salpingo-oophorectomy and SLN mapping. Among a total of 60 patients, 38 (63.3%) underwent laparoscopic surgery and 22 (36.7%) robotic surgery. We compared SLN detection rate and perioperative outcomes between the two routes of approach. **Results**: No significant differences were observed in the overall and bilateral SLN detection rate between laparoscopic and robotic surgery (97.3% and 84.2% vs. 95.5% and 91%, respectively). Significant differences were observed in operative time, with a median of 125 vs. 110 min (*p* = 0.004), and in hospital stay, with a median of 3 vs. 2 days (*p* = 0.002), with both being shorter in the robotic surgery group. No differences were observed in terms of number of SLN detected, percentage of positive nodes, intra or postoperative complications rate, or percentage of conversion to laparotomy. **Conclusions**: No differences were found in SLN detection rates between laparoscopic and robotic surgery. However, robotic surgery demonstrated advantages in terms of reduced operative time and shorter hospital stay.

## 1. Introduction

Endometrial cancer is the sixth most common malignancy among women worldwide and the second most frequent gynecologic cancer after cervical cancer [1]. In developed countries, it is the most common gynecologic malignancy [2]. The standard treatment for early-stage endometrial cancer consists of total hysterectomy with bilateral salpingo-oophorectomy and lymph node staging [2]. SLN mapping has been established as a standard alternative to full lymphadenectomy in order to reduce the risk of complications and morbidity associated with the procedure [3,4,5].

The most commonly used tracers for the sentinel lymph node detection are blue dye, technetium-99, and indocyanine green (ICG). Between them, ICG has been shown to improve detection rates and negative predictive value (NPV) compared to the other options [2].

Both laparoscopic and robotics-assisted surgical approaches have been used for SLN detection by means of fluorescent tracer, but each platform has shown differences in the visualization output, which could affect its accuracy. Robotic surgery, unlike conventional laparoscopy, provides 3D high-definition images, which significantly improves depth perception and precision. Furthermore, the surgeon directly controls the camera from the console, allowing for a much more stable image, with more complex angles and image magnifications of up to 10-15 times without a significant loss of quality, allowing for fine anatomical details to be distinguished [6]. Laparoscopic surgery also uses a high-definition camera, but the images obtained are in 2D, which limits depth perception and the ability to view complex angles. Moreover, the camera is controlled by an assistant, which can result in less stable movements depending on the assistant’s control [7].

Some studies, such as the one published by Geppert et al., have demonstrated the effectiveness of sentinel lymph node biopsy using indocyanine green in laparoscopic surgery, showing bilateral detection rates of 96% [8], which are very similar to those reported by Papadia et al., at 95.5% [9]. In robotic surgery, bilateral detection rates appear to be slightly lower, around 85.7% [10] to 87.61% [11], depending on each author.

Robotic surgery has demonstrated several perioperative advantages over laparoscopy, particularly greater surgical precision thanks to the robotic system’s ability to filter out tremors, and allowing for finer movements, which reduces the risk of damage to surrounding tissues, more careful dissection, and less perioperative bleeding, which also leads to reduced postoperative pain, a shorter hospital stay, and faster recovery [12,13,14].

Currently, there are no conclusive data demonstrating differences in sentinel lymph node detection rates using ICG between laparoscopic and robotic surgery. This study aims to compare SLN mapping rates between laparoscopic and robotic approaches in patients with early-stage endometrial cancer, as well as to evaluate the perioperative outcomes and complications between both surgical routes.

## 2. Materials and Methods

A retrospective, single-center, observational study was conducted from February 2024 to March 2025, including all patients diagnosed with stage I endometrial cancer [15] who met the inclusion criteria, and who underwent minimally invasive surgery at Hospital Universitario La Paz in Madrid, Spain.

It is important to note that robotic surgery in gynecologic oncology was introduced in our center in February 2024. All patients who underwent robotic surgery and met the inclusion criteria were selected. For comparison, the most recent patients who underwent laparoscopic surgery and met the inclusion criteria were retrospectively selected until reaching a total sample of 60 patients.

The inclusion criteria included histological confirmation of invasive disease, age over 18 years, suitability for minimally invasive surgery, and preoperative early-stage disease (FIGO stage I by preoperative imaging).

The work-up for all patients included a physical examination, endometrial biopsy by hysteroscopy, and transvaginal ultrasound. For patients with suspected FIGO stage IB, a pelvic magnetic resonance was performed to assess myometrial invasion and nodal involvement [15]. We also carried out a thoraco-abdomino-pelvic CT scan in non-endometrioid histologies or high-grade endometrioid tumors to rule out distant disease.

Patients were excluded from the final analysis if they presented either extra-uterine disease on pre-operative imaging or intra-operative complications regarding endoscopic route of approach, such as conversion to laparotomy.

All patients signed informed consent for surgery and underwent total hysterectomy with bilateral salpingo-oophorectomy and SLN mapping using ICG in all cases.

The decision to operate on patients utilizing the laparoscopic (Rubina, KARL STORZ, Tuttlingen, Germany) or robotic (Da Vinci Xi, Intuitive, Sunnyvale, CA, USA) approach depended on the robotic platform availability.

ICG solution was prepared by diluting 25 mg of ICG powder Verdye^®^ (Diagnostic Green GmbH, Munich, Germany) in 10 mL of sterile water (final dilution 2.5 mg/mL). After anesthetic induction, 4 mL (10 mg) of ICG was injected into the cervix at 3 and 9 o’clock positions; 1 mL deep (1 cm) and 1 mL superficial (3–4 mm).

**Figure 1:**
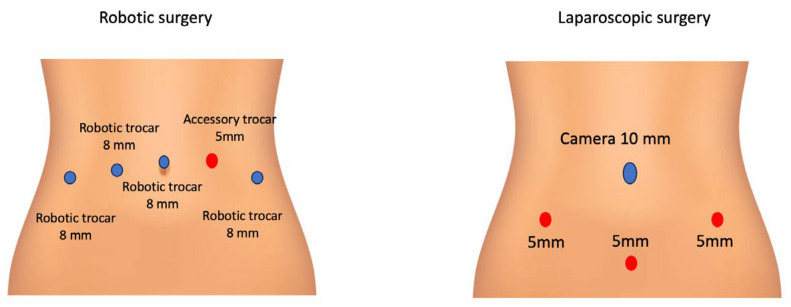
Trocar placement in robotic and laparoscopic surgery for endometrial cancer.

In robotic and laparoscopic surgery for endometrial cancer, the trocar placement was performed as indicated in Figure 1.

Uterine manipulator RUMI II^®^ (Koh Efficient, Trumbull, CT, USA) was used in all surgeries, independently of surgical approach. About 10–15 min after the cervical injection and trocar disposition, the exploration of the abdominal cavity and opening of retroperitoneal space was performed. Next, bilateral pelvic retroperitoneal evaluation and nodal assessment was performed with a near-infrared camera. The first mapped lymph node following the MSKCC algorithm was resected in each side [16]. After the performance of SLN biopsy, a total hysterectomy and bilateral salpingo-oophorectomy was carried out. When SLN was not identified, a reinjection of ICG was performed (only once reinjection is performed). Even so, if no SLN was detected on the hemipelvis, a side-specific pelvic lymphadenectomy was performed, except in cases of preoperative FIGO IA stage of low-grade endometrioid carcinoma.

No frozen sections of SLNs were performed. The sentinel lymph nodes obtained were analyzed by ultrastaging or OSNA method, depending on the cytokeratin 19 status of the tumor.

Following the final histopathological diagnosis, adjuvant treatment was administered in accordance with the recommendations outlined in the national guidelines of the Spanish Society of Gynaecology and Obstetrics (SEGO) and the international clinical guidelines of the European Society of Gynaecological Oncology (ESGO) [3,5]. All procedures were performed by the same onco-gynecologic surgical team with expertise in advanced endoscopic surgery.

The overall detection rate was defined as the percentage of patients in whom one SLN was identified on at least one side of the pelvis. The bilateral detection rate was defined as the percentage of patients with at least one SLN identified in each side of the pelvis.

Operative time was defined as the time from the first skin incision to the last skin closure in both groups. Intraoperative complications were defined as any event occurring during the surgical procedure, and postoperative complications as those recorded within 30 days after surgery.

All statistical analyses were performed using SPSS version 28.0 (IBM Corporation, Armonk, NY, USA). Standard descriptive statistics were used to evaluate the distribution of each variable. Quantitative data were presented as median and interquartile range (IQR). Categorical variables were reported as absolute values and percentages. The distribution of variables between groups was compared with Student’s *t*-test, chi-square test, Mann–Whitney U test or Fisher’s exact test, as appropriate. Logistic regression analysis was performed to perform univariate and multivariable analyses. All *p*-values reported are two-sided and alpha error was set at 5%.

## 3. Results

A total of 60 patients who underwent surgery for endometrial cancer and met the inclusion criteria were selected. Among them, 22 patients (36.7%) underwent robotic surgery and 38 (63.3%) were operated by laparoscopic approach.

The comparison of clinical, surgical, and pathological characteristics of all patients who had SLN mapping by laparoscopic or robotic approach is summarized in Table 1. No significant differences were observed between both groups in any of the variables. The median age in laparoscopic and robotic surgery groups was 63 (IQR 54–68) and 63.5 (IQR 58.8–69.8), respectively. The most common histologic subtype in both surgical groups was low-grade endometrioid endometrial carcinoma (G1), in both preoperative biopsies (laparoscopic surgery 44.7% vs. robotic surgery 59.1%) and postoperative specimens (47.4% vs. 40.9%, respectively).

The comparison of SLN detection rates and perioperative outcomes between the laparoscopy and robotic groups is shown in Table 2.

No significant differences were found in the overall SLN detection rate between laparoscopy (97.3%) and robotic surgery (95.5%). The bilateral detection rate was higher in the robotic group (91% vs. 84.2%) but also without statistically significant differences between both approaches.

In six cases (five laparoscopic and one robotic), the SLN detection was unilateral. Unilateral lymphadenectomy was performed in one case of laparoscopic low-grade endometrioid carcinoma with myometrial invasion > 50%, but the final pathology report was negative for malignancy.

Bilateral SLN detection failed in two cases (one in each group). In the robotic surgery case, re-injection of indocyanine green was attempted but was unsuccessful. This patient had adhesions secondary to previous pelvic surgery, a normal BMI, and a final FIGO stage of IIIA1. Lymphadenectomy was not performed due to a preoperative early-stage of low-grade endometrioid carcinoma (with less than 50% of myometrial infiltration). In the laparoscopic case, the patient had a preoperative high-grade, early-stage endometrial cancer, so bilateral lymphadenectomy was performed without complications and negative final pathology report.

Regarding the SLN location, the most common one in both groups was the obturator fossa (51.2% in robotic surgery and 58% in laparoscopy). The second most frequent location was the external iliac region (46.3% and 43.5%, respectively). Moreover, there were no differences in median number of SLNs mapped and retrieved between the two approaches.

Three patients (5%) had positive SLNs (two with macrometastasis and one with micrometastasis). All cases were observed in the laparoscopy group. Bulky lymph nodes were resected in three patients (5%) none of them corresponding to SLNs. One case in the robotic surgery group, and the other two cases in the laparoscopy group.

We observed shorter operation time in robotic procedures with a median reduction time of 15 min which resulted statistically significant (*p* = 0.004). Similarly, significant differences were found in the length of hospital stay in favor of robotic surgery (3 days in the laparoscopic group vs. 2 days in the robotic group; *p* = 0.002).

The observed overall complication rate was low in both groups, with no statistically significant differences either intra or postoperatively (*p* = 0.346 and *p* = 0.872, respectively). The most common intraoperative complication was uterine perforation (5%), occurring in two robotic cases and in one laparoscopic case. One bladder injury was reported during a laparoscopic procedure and one vaginal mucosal tearing occurred during a robotic surgery. Postoperative complications included two cases of vaginal cuff hematoma, one in each group. Additionally, in the laparoscopic group, there were two more postoperative complications: one case of postoperative fever and one of intestinal evisceration through a trocar site with subsequent bowel obstruction (Table 3).

No differences in conversion to open surgery were observed between surgical routes. It was required in three patients (5%) for specimen retrieval due to large myomatous uterus; one in the robotic group and two in the laparoscopic group. No conversions were due to intraoperative complications such as adhesions, bleeding, or poor visualization.

At the time of analysis, 59 patients (98.3%) were in remission, with a median follow-up of 5.5 months (IQR 2–13.8). Only one nodal paraaortic recurrence after six months of primary surgery (1.7%) was observed, in a patient treated with robotic surgery, and no deaths were recorded.

An analysis of potential variables that may affect to SLN detection is reported in Table 4. An univariate and multivariate analysis was performed which did not identify any variables with statistically significant associations.

## 4. Discussion

Currently, early-stage endometrial cancer is one of the main indications for the use of minimally invasive surgery, even for patients with high-risk endometrial cancer [3,4,17,18,19].

The results of our study have demonstrated that there were no differences between laparoscopic and robotic approach in terms of SLN detection rate in patients with early-stage endometrial cancer. Furthermore, the median number of SLN detected, number of positive nodes, or perioperative complications did not differ among both surgical approaches.

This study also has showed a high overall SLN detection rate (96.7%), in both surgical approaches using ICG (97.3% laparoscopic and 95.5% robotic surgery). The bilateral detection rate was 91% in the robotic group and 84.2% in the laparoscopic group; data comparable to those reported in the literature. A meta-analysis by Lin et al. [20] reported a bilateral detection rate of 82% for laparoscopic surgery and 86% for robotic surgery, supporting the reliability of our findings. Another meta-analysis by Raffone et al. demonstrated that both surgical approaches showed similar efficacy in SLN detection when ICG is used as the tracer with an OR of 1.80 (95% CI: 0.35–9.17) for overall detection, an OR of 1.12 (95% CI: 0.56–2.23) for bilateral detection, and an OR of 1.12 (95% CI: 0.45–1.67) for unilateral detection [21], which is also consistent with our results.

Moreover, the most frequent locations for SLNs in our study were the obturator and external iliac regions, in line with other studies identifying these areas as the most frequent sites for lymphatic spread in endometrial cancer. Chaowawanit et al. identified the external iliac region as the most frequent location (50–57.6%, depending on the surgical approach and laterality), followed by the obturator region (9.1–23.1%) [2] similar to Bizzarri, who identified the external iliac (57.3%) as the most frequent site of SLN mapping followed by the obturator fossa (30.1%) [22].

Regarding the operating time, statistically significant differences were found between both approaches (*p* = 0.004), with a median operating time of 125 min (range: 115–175) for laparoscopic surgery and 110 min (range 80–123.75) for robotic surgery. The literature findings are contradictory, with some studies favoring robotic surgery, finding statistically significant differences, such as Mäenpää et al., who reported operating times of 130 min (range: 120–499) for laparoscopic surgery and 139 min (range: 86–377) for robotic surgery (*p* < 0.001) [23]. Similarly, Coronado et al. observed statistically significant differences between laparoscopic and robotic surgery in endometrial cancer (218.2 min in laparoscopic surgery versus 189.2 min in robotic surgery, *p* < 0.0001). In both studies, pelvic lymphadenectomy was performed; however, in the case of Coronado et al., the differences remained statistically significant even when the lymphadenectomy time was excluded (169.2 min in laparoscopic surgery vs. 138.3 min in robotic surgery, *p* = 0.015) [24].

Other studies favor laparoscopy, such as the study by Perrone et al., which reported a mean operative time of 160 min (range 32–680) for laparoscopy vs. 180 min (range 50–545) for robotic surgery (*p* < 0.001). This study included patients who underwent both lymphadenectomy and sentinel lymph node biopsy [25]. In the study by Cárdenas-Goicoechea et al., lymphadenectomy was performed in all patients, with mean operative times of 170 min for laparoscopy vs. 222 min for robotic surgery (*p* < 0.0001) [26], similar to another study by the same author, which reported mean operative times of 161 min for laparoscopy vs. 218 min for robotic surgery, also showing statistically significant differences (*p* < 0.001) [27].

These inconsistencies may be due to heterogeneity in how surgical times are recorded, differences in surgeon experience, the learning curve, the use of SLN or lymphadenectomy, or procedural complexity. In our series, docking time was not included; thus, only skin-to-skin operative time was analyzed, and in most of the cases we did not perform lymphadenectomy. This metric has been reported to be significantly shorter in robotic surgery compared to total surgical time [14].

In our study, hospital stay was significantly shorter in the robotic surgery group, with a median of 2 (range: 1–2) days compared to 3 (range: 2–3) days in the laparoscopic group (*p* = 0.002). Studies involving similar populations suggest shorter hospital stays in robotic surgery, although these differences are not always statistically significant. Cárdenas-Goicoechea et al. reported mean hospital stays of 2.45 days for laparoscopic surgery vs. 1.96 days for robotic surgery, with a statistically significant difference (*p* = 0.016) [23]. Similarly, Soliman et al. described average hospital stays of 2 days for laparoscopy vs. 1 day for robotic surgery (*p* < 0.01) [25]. In the study by Gracia et al., the mean hospital stay in the robotic surgery group was 2.9 ± 2.2 days, slightly shorter than that of the laparoscopic group, which was 3.2 ± 3.8 days. However, no statistically significant differences were observed between the two groups (*p* = 0.164) [14]. These findings may be attributed to less tissue manipulation and reduced surgical trauma in robotic procedures, suggesting that robotic surgery could promote faster recovery and lower morbidity, without compromising SLN detection accuracy.

No significant differences were observed in complication rates between the two groups. These findings are consistent with previous studies. As shown in the meta-analysis by Raffone et al., which reported an OR of 0.92 (95% CI: 0.18–4.59) for intraoperative complications and an OR of 0.37 (95% CI: 0.13–1.07) for postoperative complications [19]. Similarly, Perrone et al. did not detect statistically significant differences in the rate of intraoperative (*p* = 0.133) or postoperative complications (early *p* = 0.102, late *p* = 0.734) [23]. Cárdenas-Goicoechea also found no statistically significant differences in intraoperative (*p* = 0.714) or postoperative complication rates (immediate *p* = 0.412, at discharge *p* = 0.818) [26].

In the same study, an intraoperative complication rate of 2% was reported for robotic surgery and 3.5% for laparoscopy, along with an immediate postoperative complication rate of 14.7% for robotic surgery and 19.1% for laparoscopy, and a complication rate at discharge of 8.8% for robotic surgery versus 7.5% for laparoscopy [26]. These data slightly differ from ours, since we observed a higher rate of intraoperative complications in both groups (13.6% in robotic surgery and 5.2% in laparoscopy), although the difference was not statistically significant. Conversely, the rate of postoperative complications was lower in our series (4.5% in robotic surgery and 2.6% in laparoscopy). The slightly higher incidence of complications in robotic surgery could be attributed to its recent introduction in our center. Nevertheless, due to the small sample size of our study, our results are not robust enough. Despite this, the vast majority of complications in our cohort were mild, and only one patient (1.6%) required surgical reintervention.

Other studies have associated robotic surgery with lower conversion rates to laparotomy compared to conventional laparoscopy [23,24,26], although these differences are not always statistically significant. Mäenpää et al. reported five conversions to laparotomy, all of which occurred in the laparoscopic group (*p* = 0.027) [23]. Coronado et al. reported ten conversions to laparotomy—seven in the laparoscopic group and three in the robotic group—with a non-significant difference (*p* = 0.181). Conversions were due to obesity, adhesions, or a combination of obesity and hypercapnia [24]. Cárdenas-Goicoechea et al. observed a relative risk of conversion to laparotomy between robotics-assisted and traditional laparoscopic cases of 5.3, although this difference did not reach statistical significance at a 95% confidence level (1% vs. 5.2%, *p* = 0.096) [26].

In our study, all patients underwent surgery entirely through minimally invasive approaches. Conversion to open surgery was performed solely for specimen extraction, and in all cases, this was due to a polymyomatous uterus. Although our sample size was not large enough to detect differences in this regard, these findings do not suggest any significant difference in the conversion rate between the two surgical approaches.

Finally, although it is not the focus of our study, as molecular classification is becoming increasingly central in guiding treatment strategies, we believe that integrating molecular data into staging systems may ultimately enhance risk stratification and support more personalized therapeutic decisions [28].

This study has limitations that should be acknowledged. The relatively small sample size and the unequal number of patients in each group may reduce the statistical power of the analysis. Additionally, the retrospective design makes the study more susceptible to selection and uncontrolled confounding biases. Finally, as this is a single-center study, the findings may not be generalizable to other settings with different levels of surgical expertise.

## 5. Conclusions

In conclusion, no differences were observed in SLN detection rates between laparoscopic and robotic surgery using ICG as single tracer. Therefore, our findings support that both laparoscopic and robotic approaches are valid and safe options for performing SLN mapping in endometrial cancer. While robotic surgery shows advantages in terms of shorter operative time and hospital stay.

## Figures and Tables

**Table 1:** Demographic characteristics comparing laparoscopic vs. robotic surgery.

	Laparoscopic Surgery (n = 38) Median (IQR, %)	Robotic Surgery (n = 22) Median (IQR, %)	*p*-Value
**Age (years)**	63 (54–68)	63.5 (58.75–69.75)	0.103
**BMI (kg/m^2^)**	29.5 (25–32.25)	29.5 (25.75–32)	0.829
**Previous abdominal surgery**	13 (34%)	8 (36.4%)	0.866
**Pre-operative histologic result**			0.122
- **Endometrioid**	26 (68.4%)	19 (86.4%)	0.251
**Grade 1**	17 (44.7%)	13 (59.1%)	
**Grade 2**	9 (23.7%)	5 (22.7%)	
**Grade 3**	0	1 (4.55%)	
- **Non-endometrioid**	12 (31.6%)	3 (13.6%)	
**Serous**	5 (13.2%)	2 (9.1%)	
**Clear cell**	3 (7.9%)	0	
**Others**	4 (10.5%)	1 (4.55%)	
**Post-operative histologic result**			0.184
- **Endometrioid**	27 (71.1%)	18 (81.8%)	0.380
**Grade 1**	18 (47.4%)	9 (40.9%)	
**Grade 2**	8 (21%)	7 (31.8%)	
**Grade 3**	1 (2.6%)	2 (9.1%)	
- **Non-endometrioid**	11 (28.9%)	4 (18.2%)	
**Serous**	5 (13.2%)	2 (9.1%)	
**Clear cell**	1 (2.6%)	0	
**Others**	3 (7.9%)	2 (9.1%)	
**No malignancy**	2 (5.3%)	0	
**FIGO stage (2023)**			0.658
- **Stage I:**	26 (68.4%)	15 (68.2%)	
**Ia**	23 (60.5%)	12 (54.5%)	
**Ib**	2 (5.3%)	1 (4.55%)	
**Ic**	1 (2.6%)	2 (9.1%)	
- **Stage II:**	9 (23.7%)	5 (22.7%)	
**IIa**	1 (2.6%)	0	
**IIb**	2 (5.3%)	3 (13.64%)	
**IIc**	6 (15.8%)	2 (9.1%)	
- **Stage III:**	3 (7.9%)	2 (9.1%)	
**IIIa**	0	1 (4.55%)	
**IIIb**	0	0	
**IIIc**	3 (7.9%)	1 (4.55%)	
**LVSI**			0.243
- **Absent**	29 (76.3%)	12 (54.5%)	
- **Focal**	2 (5.3%)	4 (18.2%)	
- **Present**	7 (18.4%)	6 (27.3%)	
**Myometrial invasion**			0.841
- **Absent**	14 (36.8%)	7 (31.8%)	
- **<50%**	19 (50%)	11 (50%)	
- **>50%**	5 (13.2%)	4 (18.2%)	
**Molecular study**			
- **MMRD mutated**	13 (34.2%)	7 (31.8%)	0.850
- **p53 mutated**	8 (21.1%)	3 (16.6%)	0.474
- **Pole**			0.107
**Negative**	6 (15.8%)	0	
**Mutated**	0	1 (4.5%)	
**Unknown**	32 (84.2%)	21 (45.5%)	

BMI: body mass index; FIGO: international federation of gynecology and obstetrics; LVSI: lymphovascular invasion; MMRD: mismatch repair deficiency; n: number of cases.

**Table 2:** SLN detection rate and perioperative outcomes by surgical approach. n: number of cases.

	Laparoscopic Surgery (n = 38) Median (IQR, %)	Robotic Surgery (n = 22) Median (IQR, %)	*p*-Value
**Overall SLN detection rate**	37 (97.4%)	21 (95.5%)	0.900
- **Unilateral**	5 (13.2%)	1 (4.5%)	
- **Bilateral**	32 (84.2%)	20 (91%)	0.698
- **No detection**	1 (2.6%)	1 (4.5%)	
**Median SLN detected**	2 (2–2)	2 (2–2)	0.192
**Number of positive SLN**	3 (7.8%)	0	0.292
- **Micrometastasis**	1 (2.6%)	0	
- **Macrometastasis**	2 (5.2%)	0	
**Operating time (minutes)**	125 (115–175)	110 (80–123.75)	0.004
**Length of stay (days)**	3 (2–3)	2 (1–3)	0.002
**Complication rate**	5 (13%)	4 (18%)	0.712
- **Intraoperative complications**	2 (5.2%)	3 (13.6%)	0.346
- **Postoperative complications**	3 (7.8%)	1 (4.5%)	0.900
**Conversion to open surgery**	2 (5.2%)	1 (4.5%)	0.900

**Table 3:** Intraoperative and postoperative complications by surgical approach. n: number of cases.

	Laparoscopic Surgery (n = 38) (%)	Robotic Surgery (n = 22) (%)	Total (%)
**Intraoperative complications**	2 (5.3%)	3 (13.6%)	5 (8.3%)
- Uterine perforation	1 (2.6%)	2 (9%)	3 (5%)
- Bladder injury	1 (2.6%)	0	1 (1.7%)
- Mucosal tearing	0	1 (4.5%)	1 (1.7%)
**Postoperative complications**	3 (7.9%)	1 (4.5%)	4 (6.7%)
- Vaginal cuff hematoma	1 (2.6%)	1 (4.5%)	2 (3.3%)
- Postoperative fever	1 (2.6%)	0	1 (1.7%)
- Trocar site evisceration	1 (2.6%)	0	1 (1.7%)

**Table 4:** Univariate and multivariate logistic regression analysis analyzing related factors to bilateral detection.

	Univariate Analysis		Multivariate Analysis	
**Related Factors**	**Odds Ratio (95% CI)**	***p*-Value**	**Odds Ratio (95% CI)**	***p*-Value**
**BMI**	0.892 (0.767–1.036)	0.135	0.879 (0.753–1.025)	0.101
**Age**	0.930 (0.859–1.006)	0.072	0.922 (0.850–1.001)	0.054
**Previous Abdominal Surgery**	0.267 (0.057–1.254)	0.094	0.266 (0.049–1.445)	0.125
**Surgical Approach (Laparoscopic vs. Robotics)**	1.875 (0.344–10.213)	0.467	2.184 (0.355–13.443)	0.400

## Data Availability

The raw data supporting the conclusions of this article will be made available by the authors on request.

## Abbreviations

SLN	Sentinel lymph node
ICG	Indocyanine green
NPV	Negative predictive value
SEGO	Spanish Society of Gynaecology and Obstetrics
ESGO	European Society of Gynaecological Oncology
IQR	Interquartile range
